# Multi-modality assessment and role of left atrial function as an imaging biomarker in cardiovascular disease

**DOI:** 10.1007/s10554-021-02316-x

**Published:** 2021-06-24

**Authors:** Aseel Alfuhied, Prathap Kanagala, Gerry P. McCann, Anvesha Singh

**Affiliations:** 1 Department of Cardiovascular Sciences, University of Leicester, National Institute for Health Research (NIHR) Leicester Biomedical Research Centre, Leicester, UK; 2 King Saud Bin Abdulaziz University for Health Sciences, Riyadh, Saudi Arabia; 3 Aintree University Hospital, Liverpool, UK

**Keywords:** Left atrium, Echocardiography, Cardiac magnetic resonance, Strain

## Abstract

The left atrium (LA) plays a vital role in maintaining normal cardiac function. LA volume and function have been utilised as important imaging biomarkers, with their prognostic value demonstrated in multiple cardiac conditions. More recently, there has been a sharp increase in the number of publications utilising LA strain by echocardiography and cardiac magnetic resonance (CMR) imaging. However, little is known about its prognostic value or reproducibility as a technique. In this review, we aim to highlight the conventional and novel imaging techniques available for LA assessment, using echocardiography and CMR, their role as an imaging biomarker in cardiovascular disease, the reproducibility of the techniques and the current limitations to their clinical application. We identify a need for further standardisation of techniques, with establishment of ‘normal’ cut-offs before routine clinical application can be made.

**Supplementary Information:**

The online version contains supplementary material available at 10.1007/s10554-021-02316-x.

## Introduction

Traditionally left ventricular (LV) function has been the key imaging marker of prognosis in heart disease, and LV ejection fraction (EF) cut-off points have been used in heart failure (HF) guidelines to guide therapy[1, 2]. Left atrial (LA) volume has also been recognised for its association with adverse cardiovascular outcomes in the general population [3], in those at risk of developing cardiovascular disease [4] and in multiple cardiac conditions [5, 6]. LA volume indexed to body surface area forms an integral part of LV diastolic function assessment [7] and is an essential component for the diagnostic criteria for heart failure with preserve ejection fraction (HFpEF), previously referred to as diastolic heart failure [8–10]. LA function has also attracted considerable attention as a cardiovascular imaging biomarker due to its prognostic importance [11–14] and because functional abnormalities often precede adverse LA structural remodelling and overt clinical disease [9, 15–19].

Currently, LA function is routinely evaluated using traditional 2D echocardiography derived volumetric measurements [20]. 2D echocardiography however, underestimates LA volumes compared to cardiovascular magnetic resonance (CMR) imaging, which is the gold standard for volumetric quantification [21]. LA volumes and function can also be assessed using cardiac computed tomography (CT) [22, 23] however, it is a source of ionizing radiation exposure and is not routinely utilised in clinical practice, and will not be the focus of this article. LA deformation measurement is a relatively recent technique that tracks LA phasic function and allows early detection of subclinical cardiac dysfunction, even in those with normal LA size [19]. Such techniques could overcome the limitations of volumetric assessment, which relies on geometric assumptions and loading conditions [24]. Moreover, LA strain may play an important role in classifying the degree of LV diastolic dysfunction [25], potentially eliminating the complexity in diastolic dysfunction assessment. The aim of this review is to highlight the current non-invasive imaging techniques available on echocardiography and CMR for assessing LA function, and their prognostic utility.

## Left atrial phasic function

**Fig. 1:**
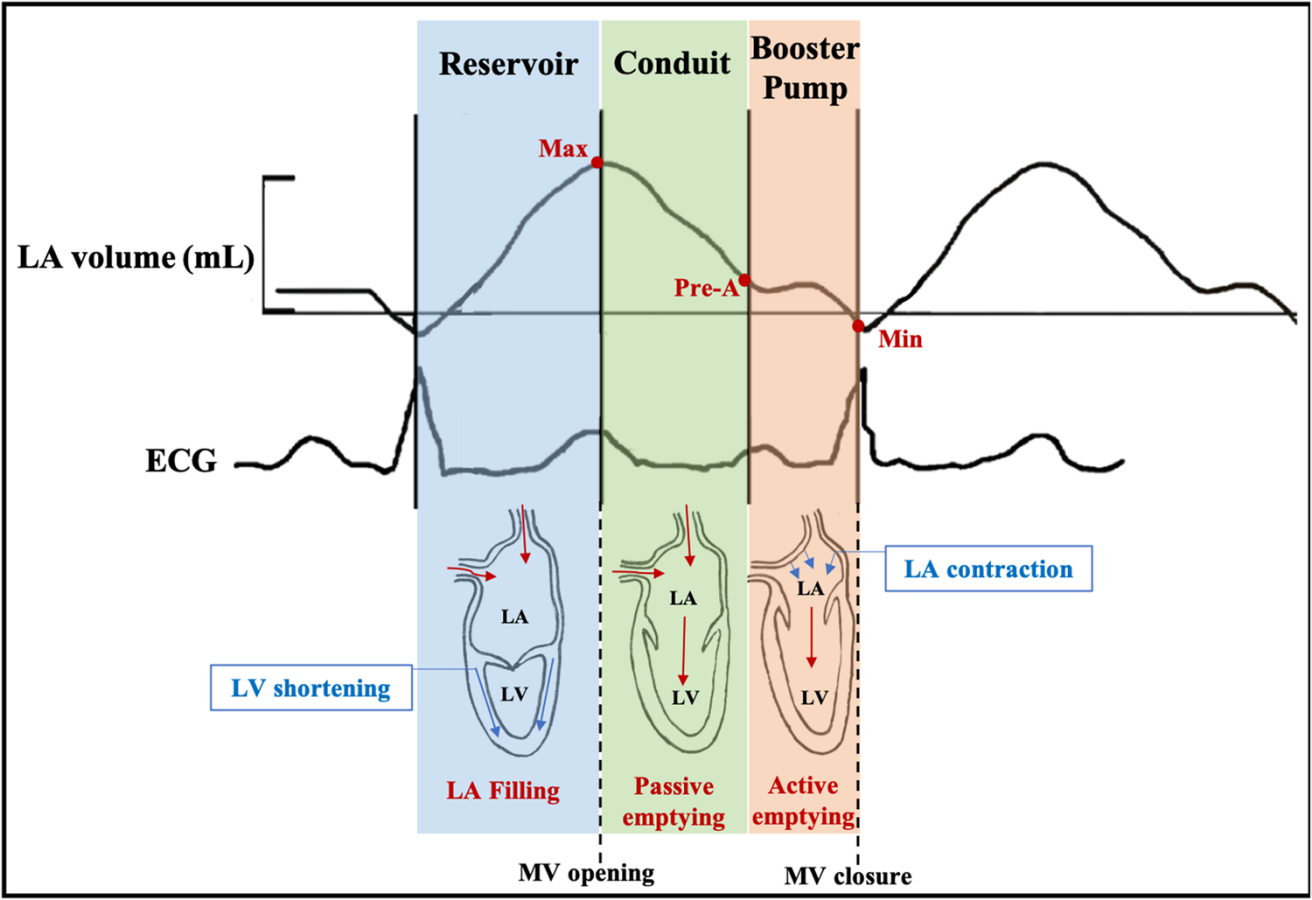
Left atrial function. Left atrial (LA) phasic function and the temporal relationship between LA volume and electrocardiogram (ECG). Pre-A = pre atrial contraction, MV = mitral valve, LV = left ventricle. Red arrows represent blood flow, blue arrows represent myocardial deformation

LA function consists of 3 phases (Fig. 1): reservoir, conduit and booster pump phases, which are responsible for the transformation of the continuous pulmonary venous return flow into intermittent LV filling [26]. During ventricular systole and isovolumetric relaxation, the LA acts as a ‘reservoir’ receiving blood flow from the pulmonary veins due to a decrease in filling pressure, leading to an increase in LA size. The conduit phase occurs during early diastole, and reflects passive emptying of the LA into the LV, governed by the transient LA to LV pressure gradient. Finally, booster pump (contraction), for those in sinus rhythm, occurs during late diastole resulting in active LA emptying attributed to the Frank-Starling mechanism, afterload and myocardial contractility [27–29].

## LA dysfunction

LA dysfunction has a marked influence on LV filling and cardiac output and is associated with the future development of HF [9]. LA reservoir function is governed by LA compliance, but is influenced by atrial contraction and relaxation, and LV systolic shortening. Thus, a decrease in atrial compliance and relaxation ability in the presence of LA stiffness causes LA reservoir dysfunction, whilst a reduction in the apical displacement of the mitral valve due to LV longitudinal dysfunction reduces passive LA stretch [30, 31].

LA conduit dysfunction results from an impairment in the atrioventricular pressure gradient mainly caused by LV diastolic dysfunction, impaired LV relaxation and increased stiffness that diminishes passive filling. With conduit impairment, the LA compensates by increasing booster pump function, which can be seen during the early stages of hypertensive heart disease [32]. However, this compensation is typically absent in patients with HFpEF due to chronically elevated LV filling pressures [33].

LA mechanical dysfunction precedes LA structural remodelling. Excessive increase in LA volume and pressure lead to histological changes such as an increase in the cardiac myocyte length, which results in progressive dilatation of the atria, myocyte hypertrophy, and fibrosis [32, 34, 35]. Moreover, LA dilatation is associated with atrial fibrillation (AF) [36]. LA booster pump dysfunction occurs in the event of abnormal LA contractility, pre-atrial contraction volume (preload), or LV end-diastolic pressure (afterload), whilst AF results in the absence of the LA booster pump function [37].

## LA functional assessment techniques

### Volumetric assessment

LA phasic function can be assessed by quantifying LA volume (LAV) in three phases across the cardiac cycle: maximum (max), minimum (min) and pre-atrial (pre-a) contraction volumes (Supplemental Figure-1). Emptying fraction (EF) is calculated corresponding to the three LA phases: **reservoir function** (LA total EF = [(LAVmax – LAVmin)/LAVmax] × 100%) and (LA expansion index =  = [(LAVmax – LAVmin)/LAVmin] × 100%) [38], **conduit function** (LA passive EF = [(LAVmax– LAVpre-A)/LAVmax] × 100%) and **booster pump function** (LA active EF = [(LAVpre-A– LAVmin)/LAVpre-A] × 100%) [16].

**Fig. 2:**
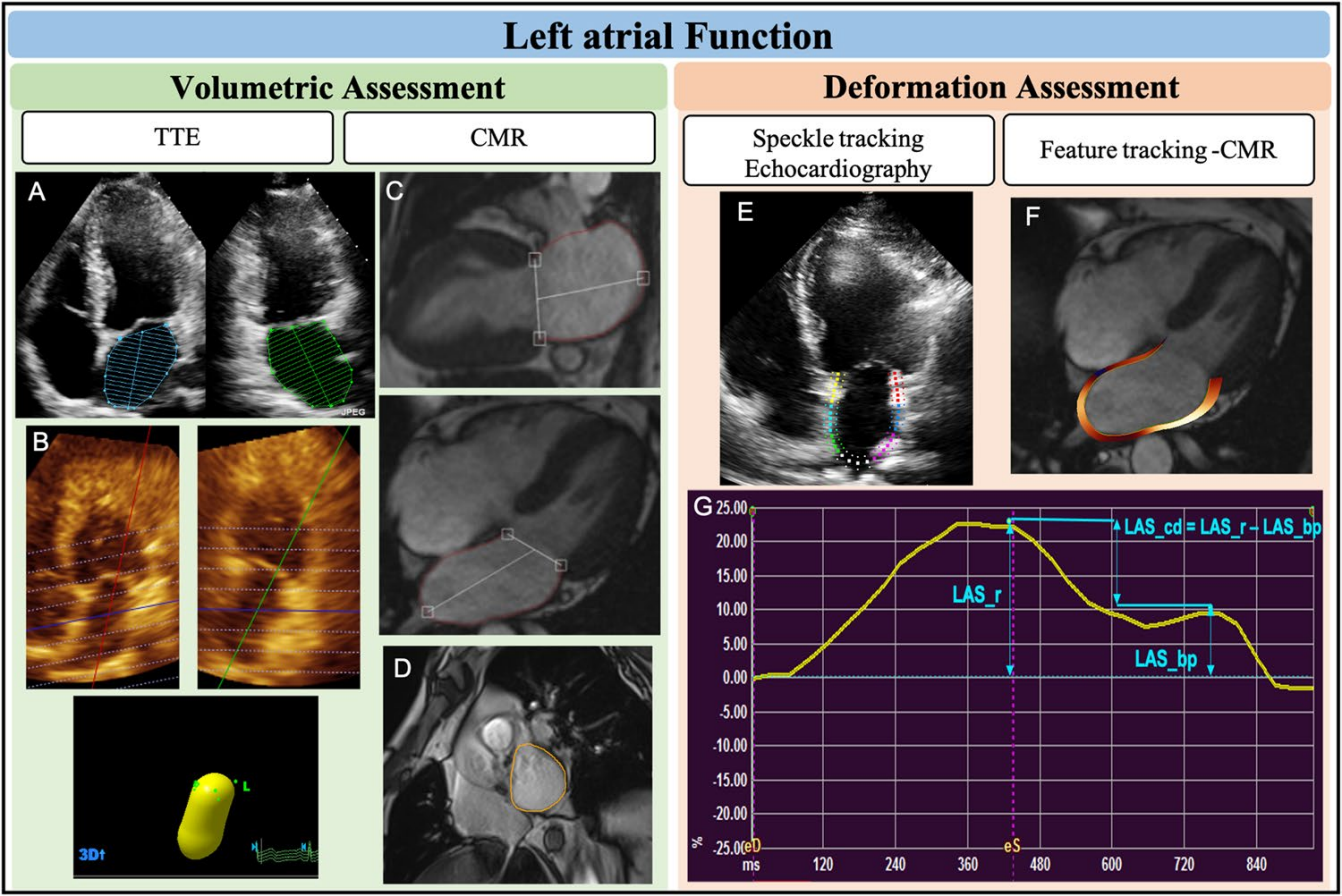
Imaging assessment of left atrial function. LA volumetric assessment using Transthoracic echocardiography (TTE) include biplane disk method (**A**) and 3D method(**B**), and using CMR include biplane area length method (**C**), and short axis stack method (**D**). LA deformation assessment using Speckle tracking echocardiography (**E**), and feature tracking on CMR (**F**). An example of LA strain curve and the measurements corresponding to LA phases (**G**). LAS_r = LA strain at reservoir, LAS_cd = LA strain at conduit, LAS_bp = LA strain at booster-pump phase

LA volume is quantified on 2D-Transthoracic echocardiography (TTE) by either the biplane area length or biplane modified Simpson’s discs method using 4- and 2-chamber images [20] (Fig. 2A). Both these methods however underestimate LA volumes compared to CMR due to variation in the spherical shape of the LA [21]. LA volumes by 3D-TTE (Fig. 2B) show better correlation with CMR than with 2D-TTE and exhibit tighter limits of agreement on Bland–Altman analysis, albeit only LAVmax showed limits of agreement within 10% [39]. On the contrary, a recent retrospective study including 56 patients in sinus rhythm showed only modest correlation and limits of agreement more than 10% for LAVmax and total EF when comparing 3D-TTE with CMR [40]. However, fully automated software was used to quantify LA volume by 3D-TTE, which may in part explain the poorer agreement.

In addition to the biplane area length method, CMR allows LA quantification using the short-axis cine stack, overcoming geometric assumptions by tracing the LA endocardial borders from successive slices across the LA length based on Simpson’s method of discs (Fig. 2C and D). LAEF by short-axis method demonstrated superior test–retest reproducibility in comparison to the biplane area-length method on CMR (CoV 4–19% and CoV 7.9–24% respectively), however, this study only included healthy volunteers and a small sample size (n = 4) [41]. The same study demonstrated no significant difference in LA volumes and EF between the two methods using steady-state free precession (SSFP) cines. Similarly, the mean LA volume was not significantly different between the two methods using SSFP images and showed excellent correlation (r = 0.92; p < 0.001), with modest agreement (-0.6 ml bias and (+ 23.5, -24.7 ml) limits of agreement) in AF patients (n = 81) [42]. In another study using the gradient-echo sequence (TrueFISP), while LA volumes were significantly higher with the biplane area-length method, there was no significant difference in LAEF between the two methods in both sinus rhythm (n = 15) and AF (n = 18) subjects [43]. Thus, despite the superior reproducibility of the short-axis method, the area-length method allows a practical and less time-consuming assessment of LA volume and function using routinely acquired 4- and 2-chamber SSFP cines, without the need for additional breath-holds for patients to acquire the short-axis cine stack.

### LA deformation assessment

Non-invasive imaging modalities have assessed LA deformation for initial diagnosis [25, 44–47], prognostic assessment [48–51], and evaluation of treatment response across different disease states [52–55]. Cardiac deformation analysis using strain and strain rate (SR) imaging allows early detection of pre-clinical cardiac disease.

Strain is an angle independent measurement that reflects the percentage of myocardial deformity (changing length) throughout the cardiac cycle. Strain is calculated as:

$$\Delta L/L0$$

where ΔL is the change in myocardial length, and L0 is the original length of the myocardium.

SR is myocardial deformity over time (the speed of myocardial deformation) [56]. Although LA strain (LAS) is preload dependent, loading has less effect on LA strain than LA volume [24], while LA strain rate (LASR) is less load-dependent than strain [57]. Both TTE and CMR use post-processing image analysis software to assess LAS and LASR, using routinely acquired 2-chamber and 4-chamber cine images. LA endocardial borders are manually traced and propagated throughout the cardiac cycle using speckle tracking in TTE or feature tracking in CMR (Fig. 2E and F).

#### Speckle Tracking Echocardiography (STE)

Tissue Doppler imaging (TDI) is an image acquisition that is used traditionally to estimates strain in TTE. However, it depends on angle of insinuation and provides regional evaluation of LA function [58, 59]. STE technique is a post-processing algorithm that quantifies LA deformation by tracking the motion of speckles within the whole myocardium through the cardiac cycle, using standard 2D echocardiography B-mode images [60](Fig. 2E). Strain and SR curves are generated after tracing the LA endocardium during systole and diastole. It is recommended to use non-foreshortened views of the LA in order to obtain adequate strain values, as well as the use of ventricular end-diastole as the time reference frame of zero strain [61].

The main limitation of STE is the need for high image quality and frame rates in order to obtain optimal endocardial tracing, which can be challenging for the LA, as it is in the far-field, and windows are affected by patient characteristics such as obesity and airways disease. This phenomenon is particularly evident in obese individuals whereby 21% of the such patients were excluded from STE analysis due to inadequate image quality [62].

#### Feature tracking CMR

Feature tracking (FT) is a post-processing strain assessment technique that uses standard CMR cine images for strain analysis (Figs. 2F and G). The analysis is performed offline using dedicated software which provides a more practical way by allowing shorter scan times. The general principle of FT is similar to STE, where features within the myocardium are tracked through the cardiac cycle. Strain assessment by CMR has several advantages over TTE such as improved spatial resolution, high signal and contrast ratio (between blood pool and myocardium), unlimited windows and clearer myocardial definition, enabling optimal tracking. Furthermore, with adequate planning, there is less propensity for foreshortened images than TTE.

Similar strain curves are generated by both TTE and CMR techniques (Fig. 2G). LAS/LASR can be measured for the three LA phases [61]: ***reservoir function*** (LAS_r and LASR_r), ***conduit function*** (LAS_cd and LASR_cd) and ***contraction booster-pump*** ( LAS_bp and LASR_bp). LAS_cd can be calculated as: LAS_cd = LAS_r – LAS_bp.

## Reference values in healthy adults

### TTE

In a population study including 371 subjects, the normal values of LA function using TomTec 2D analysis were: total LAEF 68.5 ± 5.3, passive EF 43.0 ± 10.3 and active EF 43.1 ± 9.4, while the total LAEF using 3D-TTE is 57.3 ± 4.9[63]. In a meta-analysis for normal LAS parameters by STE [64], 40 studies (2,542 patients) were included for reservoir strain, 14 studies (805 patients) for conduit and 18 studies (1,005 patients) for contractile strain, with an age range of 25–68 years. Most of the studies (n = 34) used a GE echocardiography platform (EchoPac). The normal ranges were: 27.6% to 59.8% for reservoir, 15.7% to 33.4% for conduit and 14.0% to 25.0% for booster pump strain, without significant difference between men and women. Studies on LASR normal values are limited. One study including 329 healthy adults reported the normal range of LASR at booster pump only, which was -2.11 ± 0.61 s^−1^ [65].

### CMR

Normal ranges for LAEF, LAS and LASR using CMR are shown in Table 1. With age, LA reservoir and conduit functions by volumetric assessment decrease, while booster pump function increases [66]. The LA appendage was included while the pulmonary veins were excluded from the analysis in most studies [67, 68]. Only one study focused on normal values of LA strain and strain rate by FT on 112 healthy volunteers, with a median age of 42 (IQR 30–53) years (64). The study showed no significant difference between genders in all strain and strain rate parameters. LA contractile function increased significantly with age for both strain and strain rate, while the LA conduit function decreased.

**Table 1:** The normal ranges for LA function parameters by CMR in population Studies

First Author, year (Ref. #)	Population (Male, Female)	Age	Scanner (Image analysis software)	LA function parameter	Normal range (Mean ± SD)	Comments
Hudsmith et al., 2005 [67]	HV(n = 108) (63 M, 45F)	38 ± 12 years (range 21–68)	CMR 1.5 T (Argus Siemens)	Volumetric (%): Total EF	54 ± 12%	Biplane area length method LAA included and pulmonary veins excluded from the analysis No significant difference between gender
Maceira et al., 2016 [66]	HV(n = 120) (60 M, 60F)	49 ± 17 years	CMR 1.5 T (3D-CMRTools, Cardiovascular Imaging Solutions)	Volumetric (%): Total EF Passive EF Active EF	59 ± 5.8%, 35 ± 6% 36 ± 6.8%	Data generated from 3D-modelling LAA included and pulmonary veins excluded With age LA reservoir and conduit functions decreased while the booster pump function increased Females had significantly higher conduit function than males
Petersen et al., 2017 [104]	HV(n = 795) (363 M, 432F)	59 ± 7 years (range 45–74)	CMR 1.5 T (Cvi42, version 5.1.1)	Volumetric (%): Total EF	60 ± 7%	Caucasian ethnicity only from the UK biobank Biplane area length method No significant difference between gender
Peng et al., 2018 [105]	HV(n = 150) (75 M, 75F)	43 ± 12 years	CMR 1.5 T or 3.0 T (Medis, Qmass and Qstrain)	Volumetric (%): Total EF Strain (%): Reservoir Strain	58 ± 9% 32.8 ± 9.2	Two sites: bSSFP and BTFE sequences used respectively in each site Volume by Biplane area length method Strain 2- and 4-chamber, excluding pulmonary veins and LAA No significant difference between gender Reservoir strain reduced significantly with age
Truong et al., 2019 [68]	HV(n = 112) (45 M, 67F)	42 years (median) IQR 30–53	CMR 1.5 T 2D-FT (Cvi42, version 5.3.4)	Volumetric (%): Total EF Passive EF Active EF Strain (%): Reservoir Strain Conduit Strain Contractile Strain Strain rate (s^−1^): Reservoir SR Conduit SR Contractile SR	58.8 ± 3.7 39.2 ± 6.2 31.9 ± 6.1 39.13 ± 9.27 25.15 ± 8.34 13.99 ± 4.11 1.93 ± 0.54 -2.13 ± 0.69 -2.04 ± 0.61	Volumetric by biplane area length method, LAA and pulmonary veins were excluded No significant difference between genders The LA contractile function increased significantly with age, while the LA conduit function decreased seen in both volumetric and deformation techniques
Doria de Vasconcellos et al. 2020 [106]	HV (n = 228) (91 M, 137F)	64.7 ± 8.1	CMR 1.5 T (Multimodality feature tracking version 6.0, Toshiba)	Volumetric (%): Total EF Passive EF Active EF Strain (%): Reservoir Strain Contractile Strain Strain rate (s^−1^): Reservoir SR Conduit SR Contractile SR	59.5 ± 10.5 28.2 ± 8.7 44 ± 11.3 32.6 ± 14.2 19.2 ± 9.1 1.6 ± 0.8 -1.6 ± 0.9 -2.1 ± 1.0	From Multiethnic Study of Atherosclerosis Volumetric by biplane area length method, LAA and pulmonary veins were excluded Images with poor tracking and/or foreshortened were excluded, no specific number stated No conduit strain No ethnicity comparison

*bSSFP*: Balanced Steady State Free Precession, *BTFE*: Balanced Turbo Field Echo, *FT*: feature tracking, *HV*: healthy volunteers, *LAA*: left atrial appendage, *EF*: emptying fraction, SR = strain rate

**Fig. 3:**
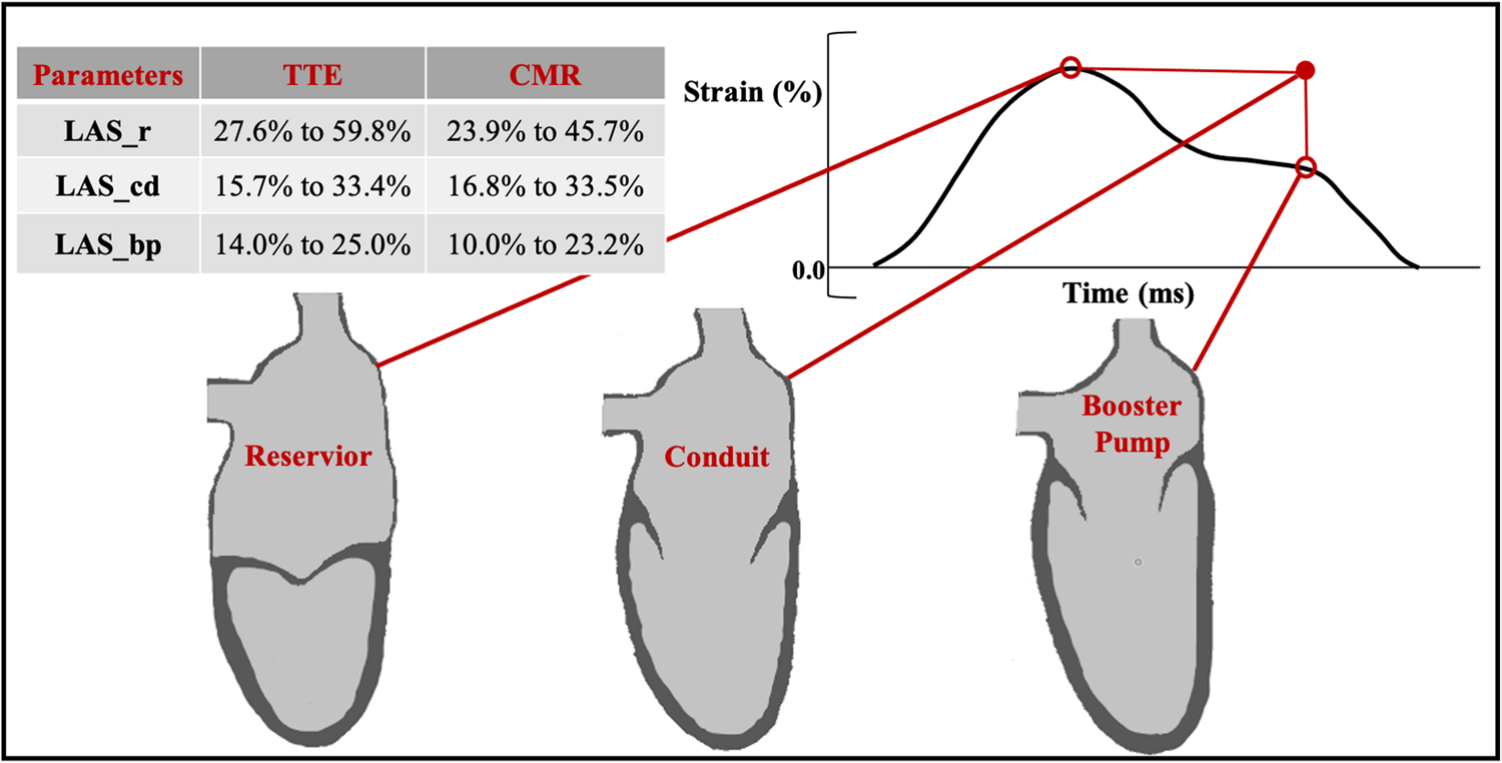
Normal values of LA phasic function by strain analysis. Table illustrates normal ranges by TTE vs CMR for: LAS_r = LA strain at reservoir, LAS_cd = LA strain at conduit, LAS_bp = LA strain at booster-pump phase. Normal ranges from[64, 68, 105, 106]. The graph illustrates the change in LA strain during the cardiac cycle

Overall, LAEF values are lower on CMR compared to TTE, but LAS values seem to be closer together (Fig. 3). The variation in values caused by vendors and imaging modality however, raises important questions regarding the validity and generalisability of the technique. Reference ranges require further validation by studying larger cohorts, and considerations of the possible influence of field strength and vendors. LASR by STE and both LAS and LASR by FT-CMR have no standardised reference ranges to date due to limited published literature on normal ranges and the variability mentioned above.

## Prognostic value of volumetric LA function

The prognostic value of LA volume [3–6, 69, 70] and size [71] is well established in multiple cardiovascular conditions. TTE studies have shown LA dysfunction, measured as a decrease in LAEF or LA function index (LAFi), to be an independent predictor of all-cause mortality or HF hospitalization in coronary artery disease [11, 72], heart failure with reduced ejection fraction (HFrEF) [73, 74] and AF[75] (Table 2). LAFi is calculated as: (LA emptying fraction × left ventricular outflow tract velocity time integral)/LA end-systolic volume index.

**Table 2:** Prognostic associations of volumetric LA function by TTE

First Author, year (Ref. #)	Population (n), Mean age	LA Parameters	Follow-up and Outcome Measure	Result
Welles et al., 2012 [11]	CAD (n = 855) 66.5 ± 10.6	2D-echo LAVI, LAEF, LAFI	HF hospitalization Median follow-up of 7.9 years	LAFI independently predictive of HF hospitalization. (HR: 1.5, 95% CI: 1.0 – 2.1; p = 0.05) in model containing all Echocardiography variables
Sargento et al., 2017 [73]	HFrEF (n = 203) 67.8 ± 12.5 years	2D-echo LAVI, LAEF, LAFI	All-cause mortality Median follow-up of 3 years	LAFI independently predictive of all-cause mortality. (HR:0.93, 95% CI: 0.89 – 0.97; p < 0.001)
Inciardi et al., 2019 [75]	AF (n = 971) 71 ± 9.4 years	2D-echo including LAEF, LAVI, LAEi	Composite of cardiovascular death or HF hospitalization Median follow-up of 2.5 years	LAEF independently predictive of composite outcome (HR:1.35, 95% CI: 1.09 – 1.67; p = 0.005) LAEi independently predictive of composite outcome. (HR:1.34, 95% CI: 1.06 – 1.69; p = 0.012) in model containing all Echocardiography variables
Modin et al., 2019 [74]	HFrEF (n = 818) 66.4 ± 11.4 years	2D-echo including LAEF, LAVI, LAVImin	All-cause mortality Median follow-up of 3.3 years	LAEF independently predictive of all-cause mortality. (HR:1.11, 95% CI: 1.01 – 1.23; p < 0.03) in model containing all Echocardiography variables
Modin et al. 2020 [72]	STEMI (n = 369) 62.2 ± 11.4 years	2D-echo including LAEF, LAVI, LAEi	Composite of all-cause mortality or HF Median follow-up of 66 months	LAEF independently predictive of composite outcome. (HR:1.25, 95% CI: 1.01 – 1.23; p = 0.043) in model containing all Echocardiography variables

*AF*: atrial fibrillation, *CAD*: coronary artery disease, *HFrEF*: heart failure with reduced ejection fraction, *HR*: hazard ratio, *LAEi*: expansion index calculated as (maximal volume–minimal volume)/minimal volume, *LAFI*: LA functional index calculated as (LA emptying fraction × left ventricular outflow tract velocity time integral)/(LA end-systolic volume index)), *LAVI:* Left atrial volume index, *STEMI*: ST-Elevation Myocardial Infarction

LAEF by CMR was recognised as a subclinical cardiac biomarker, with a decrease in LA total EF being an independent predictor of all-cause mortality and AF incidence in the general population [76, 77]. In addition, a decrease in LA active EF in patients with hypertension and no cardiovascular symptoms showed a strong predictive value for adverse cardiac events including MI, HF hospitalization and death [78].

A recent observational study demonstrated that CMR-derived LAEF using the biplane area length method was lower in patients with HFpEF compared to controls [12], and was associated with an increased risk of the composite endpoint of death and or HF hospitalization. Another study of 664 patients with HF, irrespective of LVEF, showed increasing LAEF to be independently associated with survival (HR for 10% change: 0.81, 95% CI: 0.73 –0.90), P ≤ 0.001), whereas, decreasing LAEF and increasing age predicted the incident AF [14].

## Prognostic value of LA deformation

### Speckle tracking echocardiography (STE)

LAS by STE shows a promising, non-invasive approach to predicting changes in LV filling pressure. Recent studies show that LAS at reservoir phase predicts elevated LV end-diastolic pressure in patients with coronary artery disease [79] and patients with normal LVEF [45]. Another study that included 76 patients referred for left heart catheterization demonstrated LA reservoir strain to be an independent predictor of LV filling pressure, with a cutoff value of LASr < 20% being optimal to detect elevated LV filling pressure (area under the curve 0.76) [47].

The literature highlighted the prognostic utility of LA strain as a sensitive marker to assess subclinical cardiac dysfunction [19, 80]. A recent review concluded LA strain dysfunction might precede the impairment in LV deformation in valvular disease, as it was associated with a decrease in functional capacity, even when LV global longitudinal strain was preserved, and might have a role in guiding early intervention [81]. A prospective study on 312 subjects in sinus rhythm, with known cardiovascular diseases [82], showed that LA strain during the reservoir phase using STE independently predicts cardiovascular events including AF, HF and mortality with high diagnostic accuracy (cut-off for LASr < 19%, area under the curve 0.83). LA strain showed an ability to differentiate between HF categories, independent of LA volume and other diastolic function parameters [83]. HFpEF studies have also shown LA reservoir dysfunction by strain to be independently associated with adverse outcomes and HF hospitalization [84–86].

LAS_r has also been used in calculating a surrogate of LA stiffness (calculated as the ratio of E/e’ to LAS_r) [52, 87, 88]. LA stiffness was a strong predictor of adverse outcomes (death and HF hospitalization) in a study of 215 HF patients [87].

### Feature tracking CMR

Studies assessing the prognostic value of LA strain by FT-CMR are limited. In the Multi-Ethnic Study of Atherosclerosis (MESA), LA dysfunction by FT preceded HF incidence in the asymptomatic general population, and LA reservoir strain was an independent predictor of HF[9]. The same study also concluded that LA reservoir strain independently predicted AF incidence (HR 0.68, 95% CI, 0.48–0.96) [77]. This prognostic utility has also been demonstrated in hypertrophic cardiomyopathy, where impaired LA reservoir strain (< 18%) significantly increased the risk of mortality and HF development or progression [89].

CMR studies focusing on the prognostic value of LASR are limited. Only one retrospective study with a small sample size (n = 30) showed an association between LASR during the conduit phase and the incidence of acute myocarditis, with a cut-off of -1.6 s^1−^ showing 83% sensitivity and 80% specificity [90].

## LA function as potential therapeutic target

LA function may be an important future therapeutic target and endpoint for clinical trials. A recent review summarised the mechanisms and the evaluation of LA remodelling [91], whilst another reported the relationship between LA remodelling and the development of AF and the therapeutic implications for LA remodelling reversal [92]. Studies have demonstrated LA reverse remodelling post-intervention, which was defined as an improvement in LA function [55, 93–95].

TTE-TDI has been used to demonstrate an improvement in LA contractile function post-cardioversion in AF of both short (1–6 months) [93] and chronic duration [94]. Following catheter ablation for AF, 63% of the patients demonstrated a decrease in LAV max (> 15%), accompanied by an improvement in LA longitudinal lengthening and LA shortening using TDI [96]. Similar results were reported in HF patients who underwent cardiac resynchronization therapy [95]. 2D-STE has also been utilised to assess the LA response post-intervention: LA reservoir and booster strain improved post-transcatheter aortic valve implantation at 3-month follow-up [55].

## LAS in guidelines

Despite the advantages of strain and SR, their clinical application is limited due to measurement variability. This inconsistency is related to three main factors: imaging modality, software, and operator [60]. Thus, published recommendations and guidelines in disease diagnosis that include LAS are limited due to the need for technique validation. To our knowledge, only the European Association of Cardiovascular Imaging (EACVI) and the European Heart Rhythm Association (EHRA)Expert Consensus Document on the role of multi-modality imaging for the evaluation of patients with atrial fibrillation, comments that LA lateral wall strain can be reliably imaged and LA reservoir strain < 30% indicates significant alteration of LA reservoir function, which predicts poor outcome [97].

## Inter-modality agreement

Studies that directly compare TTE and CMR in the context of LA functional assessment are limited, with the majority reporting correlation rather than agreement (Table 3). A study including 34 patients with permanent AF compared LA volumetric assessment using 2D-TTE with CMR [98]. The inter-modality correlation was moderate for volumes (*r* = 0.59 for LAmax and *r* = 0.59 for LAmin, *P* < 0.001), while poor for LAEF (*r* = 0.34, *P* < 0.05). However, the two scans were separated by 7 ± 4 days [98]. Another study in 54 patients post-myocardial infarction showed good inter-modality correlation (LAVmin r = 0.70, LAVmax r = 0.71) when scans were performed on the same day [21], though the volumes were still under-estimated by TTE. Using 3D-TTE for LA volumetric assessment also underestimates LA volume compared to the biplane area length method by CMR [40]. Moreover, whilst TTE and CMR were conducted on the same day, the agreement between the two modalities was poor by Bland–Altman analysis: LAVmax: 19.7 (-42.0 to 81.5) and LAEF: -1.6 (-28.0 to 24.9).

**Table 3:** Inter-modality agreement between TTE and CMR for LA functional assessment

First Author, year (Ref. #)	Population (n)	CMR Parameters (Image analysis software)	TTE Parameters (Image analysis software)	Finding (Reproducibility)	Comments
Kühl et al. 2012[21]	STEMI (n = 54)	1.5 T LA volumes (Argus, Siemens)	2D-TTE LA volumes (Xcelera, Phillips)	Moderate inter-modality correlation LAVmin r = 0.70, LAVmax r = 0.71	No reproducibility data for LAEF CMR and TTE on the same day LAV by CMR using short axis method. LAV by TTE using biplane area length method
Agner et al. 2013 [98]	Permanent AF (n = 34)	1.5 T or 3 T LA volumes (Argus, Siemens)	2D-TTE LA volumes (Xcelera, Phillips)	Moderate inter-modality correlation for LAV (r = 0.59, p < 0.01), and poor for LAEF (r = 0.34, p < 0.05)	CMR scans on different field strength CMR and TTE performed within 7 ± 4 days LAV by CMR using short axis method LAV by TTE using biplane area length method
Levy et al., 2019 [40]	Consecutive patients in sinus rhythm (n = 56)	3 T (Cvi42)	3D-TTE LA volumes (Dynamic HeartModel)	Moderate inter-modality correlation for LAVmax and LAEF (r = 0.65, r = 0.58 p < 0.001, respectively) Poor inter-modality agreement by BA: LAVmax: 19.7 (-42.0 to 81.5) and LAEF: -1.6 (-28.0 to 24.9)	Retrospective study Fully automated 3D-TTE analysis CMR and TTE on the same day LAV by CMR using biplane area length method and manually traced
Pathan et al. 2019[99]	Patients clinically indicate CMR (n = 43) Healthy volunteers (n = 11)	3 T CMR scanner FT by two software (Medis) (Cvi42)	2D-TTE STE by two software (EchoPac) (TomTec)	Comparing Medis vs EchoPac: Excellent inter-modality correlation (ICC = 0.90) for reservoir strain Good inter-modality correlation (ICC = 0.87) for conduit strain Modest inter-modality correlation (ICC = 0.71) for booster strain	CMR and TTE on the same day 2- and 4-chamber were used, up to 2 poorly tracked segments were excluded, if more the view not used in the analysis. Thus, the two-chamber view was excluded from the analysis in 1/54 CMR cases and 2/54 TTE cases due to poor tracking of more than two segments Modest to excellent inter-vendor correlation Scans were analysed in sequence introducing a potential bias

*AF*: atrial fibrillation, *BA*: Bland–Altman, *CoV*: coefficient of variance, *FT*: feature tracking, *ICC*: intraclass correlation, *LAEF*: left atrial emptying fraction, *LAV(max/min):* Left atrial volume (maximal/minimal), *STE*: speckle tracking echocardiography, *STEMI*: ST-Elevation Myocardial Infarction

A recent study on 43 patients with clinically indicated CMR scan and 11 healthy volunteers, compared LAS parameters by CMR and TTE. The comparison included 4 different post-processing image analysis software [99]. Overall, modest to excellent inter-modality correlation was seen, depending on which strain parameter was analysed (ICC > 0.71). Reservoir and booster strain values by STE (TomTec) were significantly higher than by FT-CMR (Medis), while conduit strain values were not significantly different. Moreover, reservoir strain had the lowest inter- and intra-observer variability for both modalities [99]. To our knowledge, no studies have evaluated inter-modality correlation for LASR.

## Reproducibility of techniques

Most studies assessing reproducibility of an imaging technique focus on inter- and intra- observer variability (Supplemental Table-1). Overall, LAS has lower inter- and intra-observer variability than LASR, and reservoir and conduit function have lower variability than booster function. However, whilst important, observer variability does not address variations in image acquisition and day-to-day physiological variation. Test–retest reproducibility of an imaging technique is fundamental for its validity and its use in longitudinal studies for monitoring disease progression or response to treatment. Studies evaluating the test–retest reproducibility of LA assessment have been limited and with small sample sizes (n = 12–22) and mainly in healthy volunteers [40, 67, 100, 101] (Table 4). However, a recent study including subjects with and without cardiovascular disease (n = 60) showed LAEF to have better test–retest reproducibility than LA strain, whilst reservoir strain accounted for the most reproducible strain parameter [102].

**Table 4:** Test–retest Reproducibility of left atrial function assessment by CMR and/or TTE

First Author, year (Ref. #)	Study Population (n)	Imaging modality (CMR or TTE)	LA assessment Parameters (Image analysis software)	Finding (Reproducibility)	Comments
Hudsmith et al. 2005 [67]	HV (n = 108) Reproducibility assessment included (n = 12)	1.5 T CMR	LAV and LAEF (Argus, Siemens)	Good test–retest reproducibility of LA total EF (Cov = 14.7%)	Scans were at least 1 week apart Biplane area length method using 2- and 4-chamber The LAA was included but the pulmonary veins were excluded
Kowallick et al. 2015 [101]	HV (n = 16) Reproducibility assessment included (n = 16)	3 T CMR	LAV (Cvi42, Circle cardiovascular imaging) LAS (TomTec)	Test–retest reproducibility was best for LAS followed by LAV then LASR LAS and LASR by 2-chamber had better test–retest reproducibility than 4-chamber LA reservoir function showed the best reproducibility for LAS, LASR, and total EF (ICC 0.94–0.97, Cov 4.5–8.2%)	3 CMR scans on the same day (at 9:00, 9:30, and 14:00) Biplane area length method using 2- and 4-chamber LAA and pulmonary veins were excluded LAS and LASR results were based on the average of tracking each view three times
Zareian et al. 2015 [100]	HV (n = 22) Reproducibility assessment included (n = 22)	1.5 T CMR	LAV & LAS (Multimodality Tissue tracking)	Modest test retest reproducibility for LA total/passive/active EF (ICC 0.48–0.57 p < 0.01), LAS and SR (ICC 0.48–0.63 p < 0.01)	CMR scans were 7–28 days apart Biplane area length method using 2- and 4-chamber LAA and pulmonary veins were excluded
Levy et al., 2019 [40]	Consecutive patients in sinus rhythm (n = 56) Reproducibility assessment included (n = 17)	3D-TTE	LAV and LA total EF (fully automated software, Dynamic HeartModel)	Test‐retest reproducibility was good for LAVmax (r = 0.91) and total EF (r = 0.80)	Retrospective study No CoV values presented, and reproducibility assessed by correlation No LAVmin Both scans performed on the same day after patient repositioning and changing the observer
Alfuhied et al., 2020 [102]	Consecutive patients in sinus rhythm (n = 54) HV (n = 6) Reproducibility assessment included (n = 60)	1.5 T and 3 T CMR	LAV, LAEF, LAS and SR (Medis)	The test–retest reproducibility was moderate to poor for all strain and strain rate parameters Strain and strain rate corresponding to reservoir phase were the most reproducible CoV = 29.9% and 28.9%, respectively The test–retest reproducibility for LAVs and LAEF was good: LAVmax CoV = 19.6% ICC = 0.89, LAVmin CoV = 27.0% ICC = 0.89 and total LAEF CoV = 15.6% ICC = 0.78	CMR scans were 7–14 days apart LAEF was calculated using the biplane area-length method Strain and volume were assessed using 4- and 2-chamber LAA and pulmonary veins were excluded

*BA*: Bland–Altman, *CoV:* coefficient of variance, *HV:* healthy volunteers, *ICC:* intraclass correlation, *LAA:* left atrial appendage, *LAEF:* left atrial emptying fraction, *LAS _bp:* LA strain at booster-pump phase, *LAS _cd*: LA strain at conduit, *LAS_r:* LA strain at reservoir, *LASR_bp:* LA strain rate at booster-pump phase, *LASR_cd*: LA strain rate at conduit, *LASR_r*: LA strain rate at reservoir, *LAV(max/min)*: Left atrial volume (maximal/minimal)

## Limitations of LA strain applications in clinical practise

Whilst LA volumes are routinely used in clinical practice, LA volumetric assessment has some limitations in assessing subclinical cardiac dysfunction, due to their lower sensitivity in assessing subtle changes and their lack of representation of myocardial contractility, as they are load-dependent measurements [103]. For LAS analysis, the anatomical characteristics of the LA, such as the thin walls and the presence of the LA appendage and pulmonary veins, make it challenging to trace the LA endocardial borders. Also, there isn’t a universal and routinely available dedicated image analysis software for LA strain analysis, and more importantly, there is a need for standardization of techniques and establishment of ‘normal’ cut-offs for the various parameters, before routine clinical application.

## Conclusions

The LA plays a vital role in maintaining normal cardiac function. Accurate LA assessment is imperative in understanding the pathophysiology of cardiovascular disease. We have reviewed the conventional and novel imaging techniques available for its assessment. Whilst these are promising and provide important insights into disease progression and add prognostic value in many conditions, there are limitations in the accurate quantification of LA function. Comparing the test–retest reproducibility of LA function assessment techniques between modalities should ideally be performed in the same cohort, in order to establish the technique with the best discriminative ability for detecting clinically relevant changes with repeated measurements.

## Supplementary Information

Below is the link to the electronic supplementary material.

Supplementary file1 (DOCX 45 KB)

Supplementary file2 (PNG 376 KB)

## Abbreviations

CMR: Cardiovascular magnetic resonance
CoV: Coefficient of variance
HF: Heart failure
LA: Left atrium
LAEF: Left atrial emptying fraction
LAS/SR_bp: Left atrial strain/strain rate at booster pump phase
LAS/SR_cd: Left atrial strain/strain rate at conduit phase
LAS/SR_r: Left atrial strain/strain rate at reservoir phase
LAV(max/min/pre-A): Left atrial volume (maximal/minimal/pre-atrial contraction)
TTE: Transthoracic echocardiography
