# Supplementary material for: Multi-modality assessment and role of left atrial function as an imaging biomarker in cardiovascular disease
Source: Int J Cardiovasc Imaging. 2021 Jun 24;37(11):3355–69. doi: 10.1007/s10554-021-02316-x (PMC8557157; doi:10.1007/s10554-021-02316-x)
Supplement: Supplementary file 1 — Supplementary file1 (DOCX 45 KB) [file 10554_2021_2316_MOESM1_ESM.docx]

**Title:**

Multi-modality assessment and role of left atrial function as an imaging biomarker in cardiovascular disease

**Names of authors and affiliations:**

Aseel Alfuhied^a,b^ – BAMSc, MSc [aa1108@leicester.ac.uk](mailto:aa1108@leicester.ac.uk)

Prathap Kanagala^a,c^– MBBS, PhD [pkk12@leicester.ac.uk](mailto:pkk12@leicester.ac.uk)

Gerry P. McCann^a^ – MBChB, MD gpm12@leicester.ac.uk

Anvesha Singh^a^ – MBChB, PhD as707@leicester.ac.uk

1. Department of Cardiovascular Sciences, University of Leicester, National Institute for Health Research (NIHR) Leicester Biomedical Research Centre, Leicester, United Kingdom.
2. King Saud bin Abdulaziz University for Health Sciences, Riyadh, Kingdom of Saudi Arabia.
3. Aintree University Hospital, Liverpool, United Kingdom

**Corresponding author details:**

Dr Anvesha Singh, Department of Cardiovascular Sciences, University of Leicester, National Institute for Health Research (NIHR) Leicester Biomedical Research Centre, Glenfield Hospital, Groby Road, Leicester LE3 9QP, United Kingdom.

E-mail: as707@leicester.ac.uk

Telephone: +44 (0)116 2044768

Fax: +44 (0)116 2583198

**Supplemental data**

**Table Supplemental 1:** **Inter- and Intra-observer variability of left atrial function assessment by CMR and/or TTE.**

| **First Author, year** (Ref. #) | **Study Population**  (n) | **Imaging modality** (CMR or TTE) | **LA assessment Parameters**  (Image analysis software) | **Finding (Reproducibility)** | **Comments** |
| --- | --- | --- | --- | --- | --- |
| Hudsmith et al. 2005 ^1^ | HV (n=108)  Reproducibility assessment included  (n=12) | 1.5T CMR | LAV and LAEF  (Argus software) | - Intra- and inter-observer variability of LA total EF ( Cov= 16.4% and Cov=9.6%, respectively).  - Variability was larger for LA comparing to LV parameters. | - Biplane area length method using 2- and 4-chamber. - The LA appendage was included but the pulmonary veins were excluded. |
| Cameli et al. 2009 ^2^ | HV (n=60)  Reproducibility assessment included  (n=20) | 2D-TTE | 2D-LAS (Reservoir)  (EchoPac) | - 4-chamber, inter- and intra-observer variability (Cov= 4.3% and 3.6%, respectively). - 2-chamber, inter- and intra-observer variability (Cov= 4.6% and 4.0%, respectively). | -Segments with inadequate tracking were excluded. |
| Saraiva et al. 2010 ^3^ | HV (n=64)  Reproducibility assessment included  (n=12) | 2D-TTE | 2D- LAS and LASR  (EchoPac) | Intra-observer variability (BA method):  LAS_r: −1.1 % (−3.1 to 0.9)  LAS_cd: −0.6 % (−1.9 to 0.7)  LAS_bp: 0.5% (−0.3 to 1.2)  LASR_r: 0.07 s^−1^ (−0.06 to 0.20)  LASR_cd:−0.06 s^−1^(−0.14 to 0.02)  LASR_bp: −0.03 s^−1^(−0.24 to 0.18)  Inter-observer variability:  LAS_r: 2.8% (0.3 to 5.3)  LAS_cd: 1.8% (0.04 to 3.5)  LAS_bp: −0.7% (−1.8 to 0.4)  LASR_r: 0.18 s^−1^ (0.09 to 0.28)  LASR_cd: −0.23 s^−1^(−0.38 to −0.09)  LASR_bp: −0.21 s^−1^ (−0.42 to −0.01) | - Retrospective study  - 2-, 4-chamber, and inferoposterior wall from 3-chamber were used.  - Observer variability assessed by BA only. |
| Kühl et al. 2012 ^4^ | STEMI (n=54)  Reproducibility assessment included  (n=20) | 1.5T CMR vs 2D-TTE | LAV  (Argus vs Xcelera software | Inter-observer variability is higher for LAVmin by TTE compared to CMR (mean percentage error 8.7±24% vs 3±10%). | - No reproducibility data for LAEF. - LAV by CMR using short-axis method, LAA included. - LAV by TTE using biplane disk method. |
| Agner et al. 2013 ^5^ | Permanent AF (n=34)  Reproducibility assessment included  (n= 20) for CMR and (n=30) TTE. | 1.5T or 3T CMR vs 2D-TTE | LAV  (Argus vs Xcelera software) | Intra and inter-observer variability for LAV were lower by CMR compared to 2D-TTE. | - No reproducibility data for LAEF. - LAV by CMR using short-axis method, LAA included.   LAV by TTE using biplane disk method. |
| Zareian et al. 2015 ^6^ | HV (n=22)  Reproducibility assessment included (n=22) | 1.5T CMR | LAV & LAS  (Multimodality Tissue tracking) | - Good to excellent intra- and inter-observer variability for LA total/ passive/ active EF, LAS_r , LASR_r and LASR_cd (ICC 0.82-0.98 p<0.001) - LASR_bp was more variable between observers (ICC=0.59 p<0.05) | - Biplane area length method using 2- and 4-chamber - LAA and pulmonary veins were excluded. |
| Dick et al. 2017 ^7^ | Acute myocarditis (n=30)  HV (n=25)  Reproducibility assessment included all subjects | Mix 1.5 and 3T  CMR | LAS and LASR  (TomTec) | LAS and SR at reservoir and conduit showed lower intra- and inter-observer variability than at booster (ICC>0.75 and Cov<13%). | - Retrospective study. - 2- and 4-chamber were used. |
| Petersen et al, 2017 ^8^ | HV (n=795)  Reproducibility assessment included (n=50) | 1.5T CMR | LAV and LAEF  (Cvi42, version 5.1.1) | - Good to excellent inter-observer variability for LAVmax and LAEF (ICC=0.96 and 0.64 respectively). - Agreement by BA for LAVmax less than 10% difference. | BA only for LAVmax no analysis for LAEF. |
| Sugimoto et al. 2018 ^9^ | HV (n=371)  Reproducibility assessment included (n=20) | 2D-TTE | 2D- LAS  2D & 3D-LAV  (TomTec) | Excellent intra-observer variability in LA reservoir strain, LA booster strain and strain rate, 3D LA volume (ICC = 0.85, 0.71, 0.79, and 0.90, P < 0.01, respectively). | - Only intra-observer variability. - Limited parameters were noted.   Both 2- and 4-chamber were used. |
| Pathan et al. 2019^10^ | Patients clinically indicate CMR (n=43)  & HV (n=11) | 3T CMR vs 2D-TTE | LAS  (Medis or Cvi42 vs EchoPac or TomTec) | - Reservoir strain had the lowest inter- and intra-observer variability for both modalities. - Intra-observer variability was the lowest for STE by TomTec (Cov=6.5%, ICC=0.95), while Inter-observer variability was the lowest for STE by EchoPac (Cov=9.9%, ICC=0.94). | - 2- and 4-chamber were used, poorly tracked segments were excluded up to two, if more the view not used in the analysis. - Strain results were based on the average of tracking each view three times for CMR, while for TTE three cine loops were analysed separately and averaged. |
| Truong et al. 2019 ^11^ | HV (n=112)  Reproducibility assessment included (n=30) | 1.5T CMR | LAS  (Cvi42) | - Good to excellent intra and inter-observer variability. - LAS had better reproducibility than LASR. - Reservoir and conduit values had better reproducibility than booster values. | - 2- and 4-chamber were used. |
| Alfuhied et al, 2020 ^12^ | 54 Consecutive patients in sinus rhythm (n=54) & HV (n=6)  Reproducibility assessment included (n=10) | 1.5T and 3T CMR | LAV, LAEF, LAS and SR  (Medis) | - LA volumetric assessment method had better inter- and intra-observer variability compared to strain assessment (Cov <19.7% vs <56.1% and Cov <12.2% vs 33.6%, respectively). | - LA volumetric assessment by biplane area length method. - 2- and 4-chamber were used. |

**Abbreviations:**

BA= Bland-Altman, CoV= coefficient of variance, HV= healthy volunteers, ICC= intraclass correlation, LAA= left atrial appendage, LAEF= left atrial emptying fraction, LAS_bp= LA strain at booster-pump phase, LAS_cd= LA strain at conduit, LAS_r = LA strain at reservoir, LASR_bp= LA strain rate at booster-pump phase, LASR_cd= LA strain rate at conduit, LASR_r = LA strain rate at reservoir, LAV(max/min) = Left atrial volume (maximal/minimal), STEMI= ST-Elevation Myocardial Infarction.

**Figures Legend**

**Figure Supplemental 1: Left atrial volume during the cardiac cycle.**

LA volume curve illustrates the change in LA volume over time. LA volumes quantified by area length method using 4-chamber CMR images. LAVmin measured at LV end-diastole, LAVmax measured at LV end-systole, and pre atrial contraction (LAVpre-A) measured at late LV diastole before atrial contraction.

**References:**

1. Hudsmith LE, Petersen SE, Francis JM, Robson MD and Neubauer S. Normal human left and right ventricular and left atrial dimensions using steady state free precession magnetic resonance imaging. *J Cardiovasc Magn Reson*. 2005;7:775-82.

2. Cameli M, Caputo M, Mondillo S, Ballo P, Palmerini E, Lisi M, et al. Feasibility and reference values of left atrial longitudinal strain imaging by two-dimensional speckle tracking. *Cardiovasc Ultrasound*. 2009;7:6-6.

3. Saraiva RM, Demirkol S, Buakhamsri A, Greenberg N, Popović ZB, Thomas JD, et al. Left Atrial Strain Measured by Two-Dimensional Speckle Tracking Represents a New Tool to Evaluate Left Atrial Function. *J Am Soc Echocardiogr*. 2010;23:172-180.

4. Kühl JT, Lønborg J, Fuchs A, Andersen MJ, Vejlstrup N, Kelbæk H, et al. Assessment of left atrial volume and function: a comparative study between echocardiography, magnetic resonance imaging and multi slice computed tomography. *Int J Cardiovasc Imaging*. 2012;28:1061-1071.

5. Agner BFR, Kühl JT, Linde JJ, Kofoed KF, Åkeson P, Rasmussen BV, et al. Assessment of left atrial volume and function in patients with permanent atrial fibrillation: comparison of cardiac magnetic resonance imaging, 320-slice multi-detector computed tomography, and transthoracic echocardiography. *Eur Heart J Cardiovasc Imaging*. 2013;15:532-540.

6. Zareian M, Ciuffo L, Habibi M, Opdahl A, Chamera EH, Wu CO, et al. Left atrial structure and functional quantitation using cardiovascular magnetic resonance and multimodality tissue tracking: validation and reproducibility assessment. *J Cardiovasc Magn Reson*. 2015;17:52.

7. Dick A, Schmidt B, Michels G, Bunck AC, Maintz D and Baessler B. Left and right atrial feature tracking in acute myocarditis: A feasibility study. *Eur J Radiol*. 2017;89:72-80.

8. Petersen SE, Aung N, Sanghvi MM, Zemrak F, Fung K, Paiva JM, et al. Reference ranges for cardiac structure and function using cardiovascular magnetic resonance (CMR) in Caucasians from the UK Biobank population cohort. *J Cardiovasc Magn Reson*. 2017;19:18.

9. Sugimoto T, Robinet S, Dulgheru R, Bernard A, Ilardi F, Contu L, et al. Echocardiographic reference ranges for normal left atrial function parameters: results from the EACVI NORRE study. *Eur Heart J: Cardiovasc Imaging*. 2018;19:630-638.

10. Pathan F, Zainal Abidin HA, Vo QH, Zhou H, D'Angelo T, Elen E, et al. Left atrial strain: a multi-modality, multi-vendor comparison study. *Eur Heart J Cardiovasc Imaging*. 2021;22:102-110.

11. Truong VT, Palmer C, Wolking S, Sheets B, Young M, Ngo TNM, et al. Normal left atrial strain and strain rate using cardiac magnetic resonance feature tracking in healthy volunteers. *Eur Heart J Cardiovasc Imaging*. 2019;21:446-453.

12. Alfuhied A, Marrow BA, Elfawal S, Gulsin GS, Graham-Brown MP, Steadman CD, et al. Reproducibility of left atrial function using cardiac magnetic resonance imaging. *Eur Radiol*. 2020:1-10.
