# Supplementary figures and images for: Multi-modality assessment and role of left atrial function as an imaging biomarker in cardiovascular disease
Source: Int J Cardiovasc Imaging. 2021 Jun 24;37(11):3355–69. doi: 10.1007/s10554-021-02316-x (PMC8557157; doi:10.1007/s10554-021-02316-x)

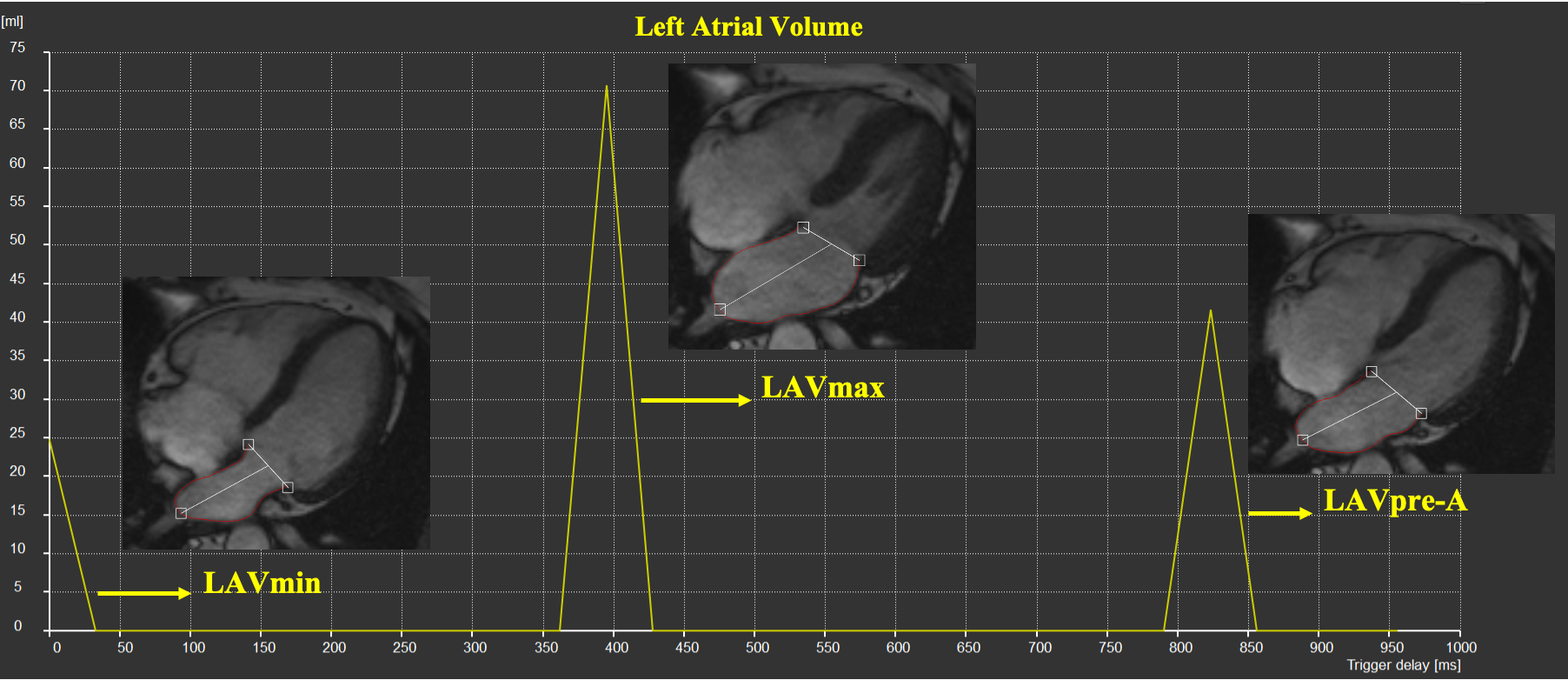

Supplement: Supplementary file 2 — Supplementary file2 (PNG 376 KB) [file 10554_2021_2316_MOESM2_ESM.png]
